# Is Mental Health Competence in Childhood Associated With Health Risk Behaviors in Adolescence? Findings From the UK Millennium Cohort Study

**DOI:** 10.1016/j.jadohealth.2020.04.023

**Published:** 2020-11

**Authors:** Emeline Rougeaux, Steven Hope, Russell M. Viner, Jessica Deighton, Catherine Law, Anna Pearce

**Affiliations:** a Population, Policy and Practice Research & Teaching Department, University College London Great Ormond Street Institute of Child Health, London, United Kingdom; b Evidence Based Practice Unit, Anna Freud Centre, University College London, London, United Kingdom; c MRC/CSO Social and Public Health Sciences Unit, University of Glasgow, Glasgow, Scotland

**Keywords:** Mental health competence, Positive mental health, Child and adolescent health, Health-risk behaviours, Lifecourse

## Abstract

**Purpose:**

Promoting positive mental health, particularly through enhancing competencies (such as prosocial behaviors and learning skills), may help prevent the development of health risk behaviors in adolescence and thus support future well-being. Few studies have examined how mental health competencies in childhood are associated with adolescent health risk behaviors, which could inform preventative approaches.

**Methods:**

Using UK Millennium Cohort Study data (n = 10,142), we examined how mental health competence (MHC) measured at the end of elementary school (11 years) is associated with self-reported use of cigarettes, e-cigarettes, alcohol, illegal drugs, antisocial behavior, and sexual contact with another young person at age 14 years. A latent measure of MHC was used, capturing aspects of prosocial behavior and learning skills, categorized as high MHC, high–moderate MHC, moderate MHC, and low MHC. Logistic and multinomial regression estimated odds ratios and relative risk ratios for binary and categorical outcomes, respectively, before and after adjusting for confounders. Weights accounted for sample design and attrition and multiple imputation for item missingness.

**Results:**

Those with low, moderate, or high-moderate MHC at age 11 years were more likely to have taken part in health risk behaviors at age 14 years compared with those with high MHC. The largest associations were seen for low MHC with binge drinking (relative risk ratio: 1.6 [95% confidence interval: 1.1–2.4]), having tried cigarettes (odds ratio: 2.2 [95% confidence interval: 1.6-3.1]) and tried illegal drugs (odds ratio: 2.0 [95% confidence interval: 1.3-3.1) after adjusting for confounders (which attenuated results but largely maintained significant findings).

**Conclusions:**

MHC in late childhood is associated with health risk behaviors in midadolescence. Interventions that increase children's MHC may support healthy development during adolescence, with the potential to improve health and well-being through to adulthood.

**Implications and Contribution**

Higher childhood mental health competence (MHC), reflecting prosocial behaviors and learning skills, is associated with lower likelihood of health risk behaviors in UK adolescents. These competencies have been improved in trials in school and early years' settings and thus hold potential for improving health and well-being across the life course.

See Related Editorial on p.625

A number of behaviors such as cigarette smoking, substance abuse, and engaging in risky sexual behaviors are associated with increased risks to health and well-being [1,2]. Although the tendency to explore different behaviors and identities may be driven by normative adolescent developmental processes, many such “health risk behaviors” are initiated during adolescence and may cluster and track into adulthood, affecting health and social outcomes such as education and employment [[2], [3], [4]]. Although the United Kingdom has made progress in reducing the prevalence of particular health risk behaviors in adolescents since the turn of the century, these remain a concern in health research and policy [3,5,6].

Positive mental health has been highlighted as a potentially important factor that may support better health and social outcomes throughout the life course [7,8]. It is a state of well-being that goes beyond the simple absence of illness or infirmity to include strengths and skills such as “efficient perception of reality, self-knowledge, exercise of voluntary control over behavior, self-esteem and self-acceptance, the ability to form affectionate relationships, and productivity” [9]. Research has demonstrated that higher levels of competencies, such as social competence, are associated with a lower prevalence of health risk behaviors in young people [[10], [11], [12], [13], [14], [15], [16]]. However, little research has examined how competencies *in childhood* are related to the development of health risk behaviors in adolescence, which could inform interventions aiming to reduce harmful behaviors and improve long-term well-being. Furthermore, competencies have seldom been explored in combination.

A multiple component competency-based conceptualization of positive mental health, referred to as mental health competence (MHC), has been developed using a competence framework describing a range of developmental tasks and abilities that children in early to midchildhood should have acquired [17,18]. This has been operationalized in early childhood in Australia [8,19,20] and in later childhood in the United Kingdom [21]. The UK-based measure of MHC captures a combination of competencies, composed of items exploring prosocial behaviors and learning skills, and has been shown to be cross-sectionally associated with emotional, cognitive, and physical health in children at age 11 years in the Millennium Cohort Study (MCS) [21]. Notably, this study found that compared with children with high MHC, those with low MHC were more likely to: report unintentional injuries on multiple occasions, be classed as obese, have two or more asthma symptoms, and have lower scores on verbal ability, spatial working memory, and risk taking tests of cognitive development [21]. Using this measure of MHC [21], we aimed to establish if MHC in midchildhood was associated with health risk behaviors in adolescence. Specifically, we examined how MHC measured at the end of elementary school (11 years) is related to health risk behaviors at age 14 years in the UK MCS.

## Methods

### Sample

Data were from the UK MCS, a nationally representative contemporary cohort of 18,818 children, born in the United Kingdom between September 2000 and January 2002, of which 18,296 were singletons first surveyed at 9 months. Subsequent surveys have been carried out at 3 (n = 15,381 singletons), 5 (n = 15,041 singletons), 7 (n = 13,681 singletons), 11 (n = 13,112 singletons), and 14 (n = 11,576 singletons) years [22,23]. Of the children who had measures of MHC at age 11 years (n = 12,082), 90% (n = 10,142) were present at the 14-year survey. Of these cohort members, 3,266 were missing data on one or more of the outcome or confounding variables; 3,265 had sufficient auxiliary information to have missing information multiply imputed. This was carried out under a missing at random assumption using multivariate imputation by chained equations in 20 datasets, providing an analytic sample of 10,141. Survey weights were used to account for sampling design and attrition at the 14-year survey.

Ethical approval was received from a Research Ethics Committee at each study survey [22,23]. Secondary data analyses do not require additional ethics approval. Further information about the MCS can be found elsewhere (http://www.cls.ioe.ac.uk/MCS). Data were downloaded from the UK Data Service, University of Essex, and University of Manchester, in May 2017.

## Measures

### Exposure: MHC at age 11 years

We used a categorical measure of MHC at age 11 years, created using a latent class analysis approach described in an earlier paper [21]. In brief, the measure was developed using maternal reports of eight positively framed questions from the validated Strengths and Difficulties Questionnaire [24], and characterizes children according to the combination of prosocial behaviors and learning skills. It consists of four classes: “high MHC” (high prosocial behaviors and learning skills), “high-moderate MHC” (high prosocial behaviors and moderate learning skills), “moderate MHC” (moderate prosocial behaviors and learning skills), and “low MHC” (moderate prosocial behaviors and low learning skills). The methods, including latent class model factors, class selection process, and analysis probability estimates, are given in more detail elsewhere [21]. In addition, the questions included in the MHC measure and latent class category probabilities are shown in Supplementary file A (Table A1).

### Outcomes: health-risk behaviors at age 14 years

Several health risk behaviors known to commonly start in early adolescence were included as outcomes.

#### E-cigarettes

E-cigarettes is a binary variable created from answers to a series of statements about e-cigarette usage, categorized as either having never used or tried e-cigarettes or having used them at least once. The latter included having used e-cigarettes in the past (prevalence: 12%), smoking e-cigarettes occasionally (3%), and smoking e-cigarettes every day (.4%).

#### Cigarettes

Cigarettes is a binary variable created from answers to a series of statements about smoking behaviors, categorized as either having never smoked cigarettes or having tried at least once. The latter includes those who have tried only once (8%), used to smoke (2%), sometimes smoke (2%), smoke one to six cigarettes a week (1%), and smoke more than six cigarettes a week (1%).

#### Alcohol consumption

A single, three-category measure of alcohol consumption was created using questions about whether the cohort member had ever consumed an alcoholic drink (more than a few sips) and if they ever had five or more alcoholic drinks at a time (a drink defined as is half a pint of lager, beer or cider, one alcopop, a small glass of wine, or a measure of spirits). It comprised having never tried drinking alcohol, having ever consumed an alcoholic drink but never tried binge drinking (defined as consuming five or more alcoholic drinks at a time; 39%), and having ever consumed an alcoholic drink including binge drinking (11%).

#### Illegal drug use

Illegal drug use is defined as having ever tried cannabis (4%) or other illegal drugs (such as ecstasy, cocaine, speed; .6%).

#### Antisocial behavior

A single binary measure of antisocial behavior captured whether the cohort member had ever engaged in one or more of the following: theft from a shop (prevalence 3%) or a person (such as a mobile phone or money 1%), graffiti (3%), public property damages (3%), carrying a knife or weapon (2%), using or hitting someone with a weapon (1%), or breaking and entering (.2%).

#### Sexual contact

Respondents were asked if they had ever kissed or cuddled another young person; if they said yes, they were asked if they had done any of the following: touched the other person's private parts (prevalence: 5%), had their private parts touched (5%), performed oral sex (2%), received oral sex (2%), and had sexual intercourse (2%). If they answered yes to any of these, we classed this as having had sexual contact.

### Confounding

We adjusted for a series of potential confounders; these were defined as measures that were associated with both the exposure and the outcomes but were not on the causal pathway between the two. Confounders that remained unchanged or stable during the time of the cohort study were assessed at 9 months: cohort member's sex, cohort member's ethnicity (white, mixed, Indian, Pakistani and Bangladeshi, Black/Black British, and other ethnic group), maternal age at cohort member's birth (14–19, 20–24, 25–29, 30–34, and 35+ years), maternal academic attainment (degree+, diploma, A levels, General Certificate of Secondary Education [GCSE] grade A∗ to C, GCSE D to G, other, and none), and any maternal smoking in pregnancy.

Confounders that were more likely to change over time were assessed at 7 years (the most recent sweep collected before both MHC and the outcomes): family structure (couple, reconstituted, or lone parent), household income (Organisation for Economic Co-operation and Development [OECD] equivalized income quintiles), maternal mental health (Kessler-6 scale [25], summed and dichotomized as none-low and moderate-severe psychological distress [26]), cohort member siblings (has any siblings in the household at age 7 years, parent report), main respondent alcohol consumption at age 7 years (self-reported number of drinks per day: never drinks, 1–2, 3–4, 5–6, 7+).

Two further confounders were also included: parent–child relationship quality, assessed only at age 3 years (using the Pianta Child-Parent Relationship Scale score, as reported by the main caregiver [27]), and puberty reported at age 11 years (using the Petersen Puberty scale [28], parental report).

### Statistical analyses

Descriptive analyses investigated associations between MHC, health risk behaviors at age 14 years, and confounders. Logistic regression was used to estimate odds ratios (ORs) for binary outcomes (cigarettes, e-cigarettes, illegal drugs, antisocial behavior, and sexual contact), and multinomial regression was used to estimate relative risk ratios (RRRs) for the three-category outcome (alcohol), according to MHC classes. We estimated ORs and RRRs (and 95% confidence intervals [CIs]) before and after adjusting for confounding. Analyses were carried out in Stata/SE 13.1 (StataCorp LP, TX).

## Results

Table 1 presents characteristics of the observed (unimputed) MCS sample, the complete case sample, and the imputed sample that was used in the main analysis. Compared with the observed MCS sample, the complete case sample was marginally less likely to display risk taking behaviors and to be from minority ethnic groups and more likely to be from a disadvantaged socioeconomic background. The analytic (imputed) sample resembled the observed MCS sample (Table 1).

**Table 1:** Prevalence of mental health competence (MHC), health-risk behaviors, and confounders in 14-year-olds in the UK Millennium Cohort Study

	Observed sample (unimputed; n = 10,142)		Complete case (n = 6,876)		Analytic (imputed) sample (n = 10,141)
	N	Weighted % (95% CI)	N	Weighted % (95% CI)	Weighted % (95% CI)
MHC at age 11 years					
High MHC (high PS; high LS)	3,997	36.4 (35.0–37.8)	2,725	37.3 (35.8–38.9)	36.4 (35.0–37.8)
High-moderate MHC (high PS; moderate LS)	3,604	36.1 (34.5–37.4)	2,521	36.8 (35.3–38.2)	36.1 (34.8–37.4)
Moderate MHC (moderate PS; moderate LS)	1,857	19.0 (18.0–20.1)	1,251	19.3 (19.2–20.4)	19.0 (17.9–20.1)
Low MHC (moderate PS; low LS)	684	8.5 (7.7–9.5)	379	6.6 (5.9–7.5)	8.5 (7.6–9.4)
Missing	1,422				
Total not at 11 year sweep (n)	5,868				
Health risk behaviors at age 14 years (CM)					
Ever consumed alcohol					
Yes but not binge drinking	3,481	38.6 (37.1–40.1)	2,602	42.2 (40.9–43.6)	38.6 (37.1–40.1)
Yes including binge drinking	923	10.6 (9.7–11.7)	642	10.3 (9.3–11.4)	10.8 (9.8–11.8)
Missing	397				
Ever tried cigarettes	1,395	16.6 (16.6–17.6)	909	15.2 (13.9–16.6)	16.9 (15.9–18.0)
Missing	431				
Ever tried e-cigarettes	1,466	17.5 (16.4–18.6)	989	17.0 (15.8–18.3)	17.8 (16.6–18.9)
Missing	426				
Ever tried illegal drugs	434	5.5 (5.0–6.2)	270	4.8 (4.1–5.5)	5.7 (5.1–6.4)
Missing	402				
Antisocial behavior	836	9.5 (8.8–10.3)	552	9.1 (8.2–10.1)	9.7 (8.9–10.5)
Missing	434				
Ever had sexual contact with another young person	530	6.2 (5.5–6.9)	358	5.8 (5.1–6.7)	6.2 (5.5–7.0)
Missing	424				
Total not at 14 year sweep (n)	7,404				
Confounders (M)					
Cohort member sex					
Male	5,063	51.8 (50.6–53.0)	3,379	50.5 (48.9–52.0)	51.8 (50.6–53.1)
Female	5,079	48.2 (47.0–49.4)	3,497	49.5 (48.0–51.1)	48.2 (47.0–49.4)
Missing	0				
Cohort member ethnicity					
White	8,305	82.9 (80.1–85.3)	6,192	90.1 (88.3–91.7)	82.9 (80.3–85.4)
Mixed	468	5.3 (4.6–6.1)	267	4.5 (3.8–5.3)	5.3 (4.6–6.1)
Indian	245	2.0 (1.5–2.6)	96	1.0 (.8–1.4)	2.0 (1.4–2.5)
Pakistani and Bangladeshi	617	4.2 (2.8–6.2)	131	1.3 (.9–2.1)	4.2 (2.5–5.8)
Black or Black British	289	3.5 (2.5–4.7)	122	2.2 (1.4–3.2)	3.5 (2.4–4.6)
Other ethnic group	217	2.2 (1.7–2.8)	68	.9 (.6–1.3)	2.2 (1.7–2.8)
Missing	1				
Maternal age at cohort member birth (years)					
14–19	602	9.8 (8.7–10.9)	370	8.6 (7.5–9.8)	9.7 (8.6–10.8)
20–24	1,592	19.0 (17.6–20.6)	926	15.6 (14.2–17.2)	19.1 (17.6–20.6)
25–29	2,735	28.3 (27.1–29.6)	1,924	28.9 (27.3–30.5)	28.3 (27.0–29.5)
30–34	3,162	27.9 (26.7–29.2)	2,374	30.8 (29.4–32.2)	27.9 (26.6–29.2)
35+	1,740	15.0 (13.9–16.1)	1,282	16.2 (15.0–17.5)	15.0 (13.9–16.2)
Missing	311				
Maternal smoking in pregnancy					
No	7,907	75.7 (74.2–77.2)	5,587	77.7 (76.1–79.2)	75.4 (73.9–76.9)
Yes	1,913	24.3 (22.8–25.8)	1,289	22.3 (20.8–23.9)	24.6 (23.1–26.1)
Missing	322				
Maternal academic attainment (at 9 months)^a^					
Degree+	2,072	15.0 (13.3–16.9)	1,607	17.8 (15.9–19.9)	15.0 (13.2–16.8)
Diploma	989	8.0 (7.4–8.7)	771	9.6 (8.8–10.4)	8.0 (7.3–8.7)
A-levels	1,031	8.6 (8.0–9.2)	791	10.1 (9.3–11.0)	8.6 (7.9–9.2)
GCSE grade A^a^-C	3,331	34.7 (33.0–36.4)	2,353	37.2 (35.3–39.2)	34.7 (33.0–36.3)
GCSE D-G	997	12.5 (11.4–13.8)	636	11.4 (10.3–12.7)	12.5 (11.3–13.7)
Other	271	2.5 (2.1–3.1)	100	1.3 (1.0–1.8)	2.5 (2.0–3.0)
None	1,428	18.7 (17.2–20.4)	618	12.5 (11.1–13.9)	18.8 (17.2–20.4)
Missing	23				
Parent–child relationship (at age 3 years)^b^					
Mean Pianta score	8,469	63.9 (63.7–64.2)	6,876	64.2 (63.9–64.4)	63.7 (63.5–64.0)
Missing	1,673				
Family structure (at age 7 years)					
Natural parents	7,203	70.0 (68.4–71.5)	5,346	72.4 (70.7–74.1)	68.3 (66.7–69.9)
Reconstituted	540	7.3 (6.6–8.2)	383	7.2 (6.4–8.0)	7.8 (6.8–8.9)
Lone parent	1,729	22.7 (21.3–24.1)	1,148	20.4 (18.9–22.0)	24.0 (22.6–25.5)
Missing	670				
Cohort member has siblings in the household (at age 7 years)					
No	1,064	11.7 (10.8–12.5)	770	11.6 (10.7–12.6)	11.5 (10.6–12.3)
Yes	8,412	88.4 (87.5–89.2)	6,106	88.4 (87.4–89.4)	88.6 (87.7–89.4)
Missing	666				
Income quintiles (at age 7 years)					
1st (highest income)	2,031	18.8 (17.0–20.7)	1,619	21.0 (19.1–23.1)	17.0 (15.2–18.7)
2nd	2,029	19.9 (18.6–21.2)	1,686	23.0 (21.7–24.5)	17.8 (16.7–19.0)
3rd	1,949	20.5 (19.2–21.9)	1,477	21.2 (19.8–22.7)	19.6 (18.3–20.9)
4th	1,807	20.5 (19.2–21.9)	1,198	18.8 (17.4–20.3)	21.7 (20.5–23.0)
5th (lowest income)	1,651	20.4 (18.7–22.1)	896	16.0 (14.6–17.4)	24.0 (22.1–25.8)
Missing	675				
Maternal mental health (at age 7 years)					
No-low distress	6,075	67.4 (66.0–68.7)	4,895	69.2 (67.7–70.6)	66.2 (64.7–67.6)
Medium/high distress	2,645	32.7 (31.3–34.0)	1,981	30.8 (29.4–32.3)	33.8 (32.3–35.3)
Missing	1,422				
Main parent's alcohol consumption (drinks per day, at age 7 years)					
Never drinks	2,337	23.5 (21.5–25.6)	1,093	16.2 (14.8–17.6)	23.6 (21.5–25.6)
1–2	4,026	39.0 (37.4–40.6)	3,140	44.1 (42.4–45.8)	39.0 (37.4–40.6)
3–4	2,214	22.6 (21.4–23.8)	1,704	24.4 (23.3–25.6)	22.5 (21.3–23.7)
5+	1,300	14.9 (13.8–16.1)	939	15.3 (14.1–16.6)	14.9 (13.8–16.1)
Missing	265				
Cohort member's level of pubertal development (at age 11 years)^b^					
Not started	333	3.2 (2.7–3.8)	224	3.2 (2.7–3.9)	3.2 (2.7–3.8)
Barely started	8,848	93.7 (93.0–94.4)	6,460	93.9 (93–94.6)	93.7 (93.0–94.4)
Definitely started	282	3.1 (2.7–3.5)	192	2.9 (2.5–3.5)	3.0 (2.6–3.5)
Missing	679				

CI = confidence interval; CM = cohort member respondent; LS = learning skills; M = main respondent; PS = prosocial behaviors.

a Supplemented with information collected at 3 years if not complete at 9 months.

b In the analyses as continuous variables.

In the analytic sample, approximately half of cohort members reported that they had tried alcohol by age 14 years, with 11% having engaged in binge drinking. Approximately 17% of cohort members had ever tried smoking cigarettes, and 18% had ever tried e-cigarettes. Trying illegal drugs, displaying antisocial behaviors, and having sexual contact with another young person were less prevalent, ranging from 6% to 10% (Table 1). Most cohort members were categorized as having high MHC (36%) or high-moderate MHC (36%) at age 11 years, with 19% and 9% as having low-moderate and low MHC, respectively.

Cohort members with low MHC were more likely to be male, have younger mothers (aged <24 years), have mothers with low (GCSE D-G) or no academic qualifications, to be in reconstituted or single-parent families, not to have siblings, to have reached puberty by age 11 years, to be in low-income families, to have mothers with moderate-severe mental distress, to have lower parent–child relationship scores, to have mothers who had smoked in pregnancy, and to have parents who drink three or more drinks on a normal drinking day (Supplementary file A, Table A2).

### MHC and health risk behaviors at 14 years

Fourteen-year-olds with lower levels of MHC (low, low-moderate, or moderate MHC) at age 11 years were overall more likely to have taken part in health risk behaviors compared with those with high MHC. The likelihood was highest in those with low MHC for all outcomes assessed with the exception of sexual contact with another young person. For example, those with low MHC had twice the relative risk of binge drinking (RRR: 2.0 [95% CI: 1.4–2.9]) and three times the odds of having tried smoking cigarettes (OR: 3.2 [2.3–4.3]). However, results also indicated that 14-year-olds with high-moderate MHC may be somewhat more likely to take part in most health risk behaviors compared with those with moderate MHC. Adjusting for potential confounding factors partially attenuated these results, but risks generally remained elevated (Table 2).

**Table 2:** Association between mental health competence (MHC) at 11 years and health risk behaviors at 14 years in the Millennium Cohort Study (imputed n = 10,141)

	High MHC (high PS; high LS)	High-moderate MHC (high PS; moderate LS)	Moderate MHC (moderate PS; moderate LS)	Low MHC (moderate PS; low LS)
Prevalence, weighted, % (95% CIs)				
Ever consumed alcohol				
No	56.0 (53.5–58.4)	46.8 (44.5–49.0)	49.7 (46.7–52.7)	46.3 (40.6–52.1)
Yes-low or moderate consumption	35.6 (33.4–37.8)	40.8 (38.7–42.9)	39.6 (36.7–42.5)	40.0 (34.4–45.6)
Yes-high consumption (binge drinking)	8.5 (7.3–9.6)	12.4 (10.9–14.0)	10.7 (8.8–12.5)	13.7 (9.8–17.6)
Ever tried cigarettes				
No	88.3 (86.9–89.6)	80.7 (79.0–82.5)	83.4 (81.1–85.7)	71.2 (66.2–76.3)
Yes	11.7 (10.4–13.1)	19.3 (17.5–21.0)	16.6 (14.4–18.9)	28.8 (23.7–33.9)
Ever tried e-cigarettes				
No	86.1 (84.6–87.7)	80.6 (78.8–82.4)	81.9 (79.4–84.3)	74.3 (69.6–79.0)
Yes	13.9 (12.3–15.4)	19.4 (17.6–21.2)	18.1 (15.7–20.6)	25.7 (21.0–30.5)
Ever tried illegal drugs				
No	96.0 (95.3–96.7)	93.4 (92.2–94.5)	94.8 (93.3–96.4)	89.8 (86.6–93.1)
Yes	4.0 (3.3–4.8)	6.6 (5.5–7.8)	5.2 (3.7–6.7)	10.2 (6.9–13.4)
Antisocial behavior				
No	92.6 (91.5–93.7)	89.7 (88.4–91.0)	89.6 (88.4–91.0)	84.2 (87.5–91.7)
Yes	7.4 (6.3–8.5)	10.3 (9.0–11.6)	10.4 (8.4–12.5)	15.8 (11.9–19.7)
Ever had sexual contact with another young person				
No	95.1 (94.2–96.0)	93.0 (91.8–94.2)	92.8 (91.4–94.3)	93.3 (90.9–95.7)
Yes	4.9 (4.0–5.9)	7.0 (5.8–8.2)	7.2 (5.7–8.6)	6.7 (4.3–9.1)
Unadjusted regression results				
Relative risk ratios (95% CIs)				
Ever consumed alcohol				
No	-	-	-	-
Yes-low or moderate consumption	-	1.4 (1.2–1.6)	1.3 (1.1–1.5)	1.3 (1.0–1.7)
Yes-high consumption (binge drinking)	-	1.7 (1.4–2.1)	1.4 (1.1–1.8)	2.0 (1.4–2.9)
Odds ratios (95% CIs)				
Ever tried cigarettes				
No	-	-	-	-
Yes		1.8 (1.5–2.1)	1.5 (1.2–1.8)	3.2 (2.3–4.3)
Ever tried e-cigarettes				
No	-	-	-	-
Yes	-	1.5 (1.3–1.8)	1.4 (1.1–1.7)	2.2 (1.6–2.9)
Ever tried illegal drugs				
No		-	-	-
Yes		1.7 (1.3–2.3)	1.3 (.9–1.8)	2.8 (1.8–4.2)
Antisocial behavior				
No	-	-	-	-
Yes	-	1.4 (1.2–1.8)	1.4 (1.1–1.8)	2.5 (1.7–3.5)
Ever had sexual contact with another young person				
No	-	-	-	-
Yes	-	1.4 (1.1–1.9)	1.5 (1.1–2.0)	1.4 (1.0–2.2)
Adjusted regression results^a^				
Relative risk ratios (95% CIs)				
Ever had an alcoholic drink				
No	-	-	-	-
Yes-low or moderate consumption	-	1.2 (1.1–1.4)	1.1 (1.0–1.3)	1.3 (.9–1.7)
Yes-high consumption (binge drinking)	-	1.4 (1.1–1.8)	1.2 (1.0–1.6)	1.6 (1.1–2.4)
Odds ratios (95% CIs)				
Ever tried cigarettes				
No	-	-	-	-
Yes	-	1.5 (1.3–1.8)	1.3 (1.0–1.6)	2.2 (1.6–3.1)
Ever tried e-cigarettes				
No	-	-	-	-
Yes	-	1.2 (1.0–1.5)	1.1 (.9–1.4)	1.4 (1.0–2.0)
Ever tried illegal drugs				
No	-	-	-	-
Yes	-	1.4 (1.1–1.9)	1.1 (.7–1.6)	2.0 (1.3–3.1)
Any antisocial behavior				
No	-	-	-	-
Yes	-	1.2 (1.0–1.5)	1.2 (.9–1.5)	1.9 (1.3–2.7)
Ever had sexual contact with another young person				
No	-	-	-	-
Yes	-	1.2 (1.0–1.6)	1.3 (.9–1.8)	1.1 (.7–1.7)

CI = confidence interval; LS = learning skills; PS = prosocial behaviors.

a Adjusted for cohort member (CM) sex, CM ethnicity, maternal age at cohort member's birth, income at 7 years, maternal academic attainment at 9 months, family structure at 7 years, parent–child relationship at 3 years, maternal mental health at 7 years, having siblings at 7 years, main parent respondent alcohol consumption (drinks per day at 7 years), smoking in pregnancy, and puberty at 11 years.

Analyses were repeated in the observed (unimputed) and complete case samples (not shown). For all outcomes, the results were very similar with patterns remaining the same as those found in the unadjusted results for the imputed sample (reported in Table 2 and the results above).

## Discussion

A measure of MHC reflecting learning skills and prosocial behaviors in late childhood was associated with health risk behaviors at age 14 years. Those with low MHC, comprising 9% of the sample, were more likely to take part in the health risk behaviors assessed with the exception of sexual contact (which covered a range of sexual behaviors). The results nonetheless indicated a nonlinear association between MHC and health risk behaviors, with a slightly higher likelihood of taking part in health risk behaviors among those with high-moderate MHC compared with moderate MHC. Particular skills or competencies may play different roles in the development of health risk behaviors in adolescence. More research is needed, however, to disentangle these patterns. Overall although results were attenuated, significant differences remained after adjusting for confounders.

A number of studies have explored the association between similar aspects of competence as those included in our measure of MHC and adolescent health risk behaviors [[10], [11], [12], [13], [14], [15], [16]], although all focused on competence in adolescence (not childhood) and none were UK-based. Several cross-sectional studies have shown that higher levels of prosocial behaviors were associated with lower prevalence of health risk behaviors, such as antisocial behavior, illegal drug use, and sexual contact [[11], [12], [13], [14]]. Cross-sectional evidence of an association for problem solving (which relates to critical thinking learning skills) and health risk behaviors was, however, mixed [[11], [12], [13]]. Three studies used longitudinal data to investigate temporal associations between aspects of competence and health risk behaviors. Of these, one found that social and learning competence (as a combined measure) in early adolescence was associated with decreased involvement with deviant peers in later adolescence and less delinquency in adulthood [15]. Two other studies found that lower social skills in early and mid-adolescence were associated with self-reported antisocial behavior in later adolescence and higher illegal drug use in adulthood, respectively [10,16].

In terms of prevalence of health risk behaviors in the United Kingdom, no comparable data were identified. Although figures have been reported for some health risk behaviors, these have used different measures of assessment, in other ages groups, or did not cover the whole of the United Kingdom [[29], [30], [31]].

### Strengths and limitations

We have examined, for the first time in the United Kingdom, the relationship between childhood MHC and adolescent health risk behaviors. There is a paucity of research exploring the association between competencies in childhood and adolescent health behaviors, particularly using combinations of competencies. Our study aimed to address this gap, using contemporary, UK-representative longitudinal data, a range of important health risk behaviors, and a measure of MHC that captured a number of mental health competencies, broadly reflecting prosocial behaviors and learning skills. The range of information available in the MCS enabled us to account for a wide range of possible confounding factors. The majority of the health behaviors examined were unlikely to have been present at age 11 years, thus minimizing the possibility of reverse causality. Sample design and attrition were accounted for with survey weights, and item missingness using multiple imputation and comparisons between complete case and imputed and nonimputed datasets showed similar patterns. The availability of questionnaire items relevant to MHC was relatively limited, only allowing the examination of two domains of MHC to be assessed (prosocial behaviors and learning skills). Nevertheless, these are important components of MHC, which may be amenable to change through intervention and as such are the focus of early years' programs [32]. The MHC items and the health risk behaviors were generated from self-reported maternal and cohort member response, respectively, which could have been affected by response bias because of influences of socioeconomic and contextual factors, memory, health, social desirability, or, in the case of the health risk behavior outcomes, the sensitive nature of the questions [33,34]. The health risk behaviors were, however, completed by the cohort members on computer devices, ensuring anonymity and privacy, and this should have minimized the risk of bias [35,36]. Our research aimed to assess whether MHC was associated with behaviors that may potentially lead to subsequent harms to health and well-being. A range of behaviors was explored and where possible, with varying levels of risk (e.g., we were able to differentiate binge drinking from lower alcohol consumption). We were not, however, able to do this for all outcomes because of the low prevalence of more extreme behaviors (e.g., regular smoking [2%] or having had sexual intercourse by age 14 years [2%]), as this did not allow sufficient power to carry out analyses by level of MHC. The measures used may, therefore, not all have equal implications in the longer term.

### Implications for policy and research

Our research shows that MHC at the end of elementary school is associated with a number of health risk behaviors at age 14 years, particularly for those with the lowest levels of MHC. Although health risk behaviors may reflect normative adolescent development, behaviors such as smoking, illegal drug use, binge drinking, antisocial behavior, and sexual risk taking are important predictors of later health risk behaviors and/or may have adverse consequences for later health (including greater likelihood of cardiovascular disease and cancer) and social well-being [[1], [2], [3], [4],[37], [38], [39]]. Research has also suggested that health risk behaviors in adolescence are likely to cluster and covary and may influence the development of more extreme or new behaviors over time [2]. It has also been found that early onset of smoking and sexual intercourse compared with peers is associated with greater involvement in risky behaviors in early adulthood [2,4]. School-based life skills and positive youth development programs may help reduce the likelihood of certain health risk behaviors, such as alcohol and drug consumption in young adolescents [2,38,[40], [41], [42]]. Prosocial behaviors and learning skills are not only amenable to intervention, but their improvement may also offer wider benefits on well-being and life chances [2,9,42]. However, more research is needed to explore the link between MHC and health risk behaviors as adolescents transition into adulthood, and more widely, in different countries and cultures, and at different periods. MHC provides a measure of positive mental health which can easily be reported by parents, teachers, or young people using data from existing surveys measured from infancy through to adolescence. As the MCS cohort members age, future work should track changes in MHC and health risk behaviors over time.

## Supplementary Data

Tables A1 and A2
