# Supplementary material for: Is Mental Health Competence in Childhood Associated With Health Risk Behaviors in Adolescence? Findings From the UK Millennium Cohort Study
Source: J Adolesc Health. 2020 Nov;67(5):677–84. doi: 10.1016/j.jadohealth.2020.04.023 (PMC7592122; doi:10.1016/j.jadohealth.2020.04.023)
Supplement: Tables A1 and A2 [file mmc1.docx]

**SUPPLEMENTARY FILE A**

| **Table A1. Latent class analysis probability estimates of classes of mental health competence (MHC) in the UK Millennium Cohort Study at 11 years, maternal report (N=10142)** | | | | |
| --- | --- | --- | --- | --- |
|  | High MHC (Hi PS; Hi LS) | High-Moderate MHC (Hi PS; Mod LS) | Moderate MHC (Mod PS; Mod LS) | Low MHC (Mod PS; Low LS) |
| Low mental health competence response | | | | |
| Considerate (child is considerate of other people's feelings) - *not true* (PS) | 0.022 | 0.018 | 0.012 | 0.201 |
| Shares (child shares readily with other children [treats, toys, pencils etc.]) - *not true* (PS) | 0.017 | 0.011 | 0.026 | 0.196 |
| Helpful (child is helpful if someone is hurt, upset or feeling ill) - *not true* (PS) | 0.010 | 0.005 | 0.024 | 0.121 |
| Kind (child is kind to younger children) - *not true* (PS) | 0.005 | 0.002 | 0.013 | 0.073 |
| Volunteers (child often volunteers to help others [parents, teachers, other children]) - *not true* (PS) | 0.006 | 0.011 | 0.050 | 0.203 |
| Thinks (child thinks things out before acting) - *not true* (LS) | 0.006 | 0.112 | 0.090 | 0.795 |
| Tasks (child sees tasks through to the end) - *not true* (LS) | 0.017 | 0.129 | 0.125 | 0.644 |
| Obedient (child is generally obedient, usually does what adults request) - *not true* (LS) | 0.016 | 0.022 | 0.034 | 0.303 |
| Moderate mental health competence response | | | | |
| Considerate - *somewhat true* (PS) | 0.038 | 0.123 | 0.557 | 0.642 |
| Shares - *somewhat true* (PS) | 0.060 | 0.130 | 0.519 | 0.562 |
| Helpful- *somewhat true* (PS) | 0.031 | 0.027 | 0.482 | 0.474 |
| Kind - *somewhat true* (PS) | 0.014 | 0.023 | 0.333 | 0.394 |
| Volunteers - *somewhat true* (PS) | 0.110 | 0.298 | 0.636 | 0.536 |
| Thinks - *somewhat true* (LS) | 0.302 | 0.846 | 0.789 | 0.153 |
| Tasks - *somewhat true* (LS) | 0.225 | 0.687 | 0.647 | 0.300 |
| Obedient - *somewhat true* (LS) | 0.105 | 0.398 | 0.649 | 0.607 |
| High mental health competence response | | | | |
| Considerate - *certainly true* (PS) | 0.939 | 0.859 | 0.430 | 0.157 |
| Shares - *certainly true* (PS) | 0.923 | 0.859 | 0.455 | 0.242 |
| Helpful - *certainly true* (PS) | 0.959 | 0.968 | 0.494 | 0.405 |
| Kind - *certainly true* (PS) | 0.981 | 0.975 | 0.653 | 0.533 |
| Volunteers - *certainly True* (PS) | 0.884 | 0.691 | 0.314 | 0.261 |
| Thinks - *certainly true* (LS) | 0.691 | 0.042 | 0.121 | 0.052 |
| Tasks - *certainly true* (LS) | 0.758 | 0.183 | 0.228 | 0.057 |
| Obedient - *very true* (LS) | 0.878 | 0.580 | 0.318 | 0.090 |

PS=prosocial, LS=learning skills

| **Table A2. Characteristics of 14 year olds in the UK Millennium Cohort Study by mental health competence (MHC, maternal report at age 11 years), % (n) or [mean]** | | | | | |
| --- | --- | --- | --- | --- | --- |
|  | High MHC (High PS; High LS) | High-Moderate MHC (High PS; Moderate LS) | Moderate MHC (Moderate PS; Moderate LS) | Low MHC (Moderate PS; Low LS) | Total |
| **Cohort member sex** | | |  |  |  |
| Male | 42.1 (1641) | 52.8 (1866) | 61.1 (1100) | 68.2 (456) | 51.8 (5063) |
| Female | 57.9 (2356) | 47.2 (1738) | 38.9 (757) | 31.8 (228) | 48.2 (5079) |
| *Missing (n)* | *0* | | | | |
| **Cohort member ethnicity** | | |  |  |  |
| White | 80.6 (3207) | 85.0 (3040) | 83.6 (1516) | 81.6 (542) | 82.9 (8305) |
| Mixed | 4.8 (181) | 5.3 (156) | 6.2 (98) | 5.5 (33) | 5.3 (468) |
| Indian | 2.3 (112) | 1.5 (77) | 2.0 (40) | 2 (16) | 2 (245) |
| Pakistani & Bangladeshi | 5.3 (270) | 3.0 (167) | 4.3 (128) | 3.6 (52) | 4.2 (617) |
| Black or Black British | 4.4 (136) | 3.0 (96) | 2.0 (36) | 4.9 (21) | 3.5 (289) |
| Other Ethnic group | 2.5 (91) | 2.1 (68) | 1.9 (39) | 2.4 (19) | 2.2 (217) |
| *Missing (n)* | *1* | | | | |
| **Maternal age at cohort member birth (years)** | | |  |  |  |
| 14-19 | 6.8 (154) | 10.6 (240) | 11.2 (131) | 15.9 (77) | 9.8 (602) |
| 20-24 | 14.5 (510) | 19.4 (588) | 20.6 (318) | 33.9 (176) | 19 (1592) |
| 25-29 | 28.3 (1050) | 29.1 (1013) | 28.1 (482) | 25.6 (190) | 28.3 (2735) |
| 30-34 | 32.3 (1382) | 27.2 (1083) | 25.9 (556) | 17 (141) | 27.9 (3162) |
| 35+ | 18.2 (783) | 13.8 (580) | 14.2 (306) | 7.7 (71) | 15 (1740) |
| *Missing (n)* | *311* | | | | |
| **Maternal academic attainment (at 9 months)** | | |  |  |  |
| Degree+ | 20.9 (1056) | 11.5 (584) | 14.4 (360) | 6.4 (71) | 15 (2071) |
| Diploma | 8.9 (395) | 8.0 (376) | 8.2 (177) | 3.8 (37) | 8 (985) |
| A-levels | 9.1 (414) | 8.6 (368) | 8.7 (197) | 5.7 (52) | 8.6 (1031) |
| GCSE grade A*-C | 32.5 (1200) | 37.9 (1287) | 34.2 (615) | 31 (226) | 34.6 (3328) |
| GCSE D-G | 9.8 (313) | 12.7 (387) | 13.4 (179) | 21.8 (118) | 12.5 (997) |
| Other | 3.4 (136) | 2.2 (77) | 1.8 (39) | 1.4 (16) | 2.5 (268) |
| None | 15.5 (471) | 19.1 (519) | 19.3 (281) | 29.9 (157) | 18.8 (1428) |
| *Missing (n)* | *23* | | | | |
| **Family structure (at 7 years)** | | |  |  |  |
| Natural parents | 77.5 (3074) | 68.1 (2476) | 67.2 (1281) | 50.5 (372) | 70.0 (7203) |
| Reconstituted | 4.8 (138) | 7.7 (207) | 7.7 (117) | 16.8 (78) | 7.3 (540) |
| Lone parent | 17.8 (527) | 24.2 (695) | 25.1 (344) | 32.8 (163) | 22.7 (1729) |
| *Missing (n)* | *670* | | | | |
| **Cohort member has siblings in the household (at 7 years)** | | |  |  |  |
| No | 9.5 (363) | 14.1 (443) | 10.9 (178) | 12.4 (80) | 11.7 (1064) |
| Yes | 90.5 (3377) | 85.9 (2937) | 89.1 (1565) | 87.6 (533) | 88.4 (8412) |
| *Missing (n)* | *666* | | | | |
| **Cohort member’s level of pubertal development (at age 11)**** | | |  |  |  |
| Not started | 3.6 (152) | 2.9 (105) | 2.7 (52) | 3.9 (24) | 3.2 (333) |
| Barely started | 93.4 (3474) | 94.4 (3203) | 93.9 (1613) | 91.9 (558) | 93.7 (8848) |
| Definitely started | 3.0 (120) | 2.8 (88) | 3.4 (53) | 4.2 (21) | 3.1 (282) |
| *Missing (n)* | *679* | | | | |
| **Income quintiles (at 7 years)** | | |  |  |  |
| 1^st^ (highest income) | 23.8 (972) | 17.3 (663) | 15.7 (324) | 9.5 (72) | 18.8 (2031) |
| 2^nd^ | 21.8 (862) | 19.7 (695) | 20.4 (388) | 10.5 (84) | 19.9 (2029) |
| 3^rd^ | 19.8 (743) | 21.6 (734) | 21.2 (365) | 17.2 (107) | 20.5 (1949) |
| 4^th^ | 18.0 (604) | 20.9 (688) | 20.9 (341) | 29.5 (174) | 20.5 (1807) |
| 5^th^ (lowest income) | 16.6 (556) | 20.5 (596) | 21.8 (323) | 33.3 (176) | 20.4 (1651) |
| *Missing (n)* | *675* | | | | |
| **Maternal mental health (at 7 years)** | | |  |  |  |
| No-low distress | 74.4 (2607) | 66.9 (2157) | 63.4 (1041) | 46.0 (270) | 67.4 (6075) |
| Med/high distress | 25.6 (823) | 33.1 (983) | 36.6 (566) | 54.0 (273) | 32.7 (2645) |
| *Missing (n)* | *1422* | | | | |
| **Parent-child relationship (at 3 years)**** | | |  |  |  |
| Mean Pianta score | [66.0] | [63.7] | [62.2] | [59.1] |  |
| *Missing (n)* | *1673* | | | | |
| **Maternal smoking in pregnancy** | | |  |  |  |
| No | 83.0 (3349) | 71.6 (2698) | 75.0 (1412) | 63.0 (448) | 75.7 (7907) |
| Yes | 17.0 (525) | 28.4 (805) | 25.0 (377) | 37.0 (206) | 24.3 (1913) |
| *Missing (n)* | *322* | | | | |
| **Main parent’s alcohol consumption (drinks per day, at 11 years)** | | |  |  |  |
| Never drinks | 24.1 (956) | 22.2 (763) | 21.9 (414) | 30.1 (204) | 23.5 (2337) |
| 1-2 | 44.4 (1746) | 36.7 (1381) | 38.3 (702) | 26.7 (197) | 39.0 (4026) |
| 3-4 | 21.1 (834) | 23.5 (839) | 23 (398) | 24.2 (143) | 22.6 (2214) |
| 5+ | 7.7 (284) | 12.9 (399) | 11.9 (202) | 13.0 (73) | 10.9 (958) |
| *Missing (n)* | *265* | | | | |
| **TOTAL** | **36.4 (3997)** | **36.1 (304)** | **19.0 (1857)** | **8.5 (684)** | **100 (10142)** |

*Supplemented with information collected at 3 years if not complete at 9 months

**In the analyses as continuous variables

PS=prosocial, LS=learning skills
